# Comparison Between NIST and AF Laser Energy Standards Using High Power Lasers

**DOI:** 10.6028/jres.112.021

**Published:** 2007-10-01

**Authors:** Xiaoyu Li, Thomas Scott (Retired), Chris Cromer, David Cooper, Steven Comisford

**Affiliations:** National Institute of Standards and Technology, Boulder, CO 80305; Air Force Primary Standards Laboratory, Heath, OH 43056

**Keywords:** BB calorimeter, beamsplitter, chopper wheel, comparison, high-energy laser, K-series calorimeter, monitor detector, responsivity, transfer standard

## Abstract

We report the results of a high-energy laser calorimeter comparison conducted by the National Institute of Standards and Technology (NIST), Boulder, Colorado and the U.S. Air Force Primary Standards laboratory (AFPSL), Heath, Ohio. A laser power meter, used as a transfer standard, was calibrated at each laboratory, sequentially, and the measurement results were compared. These measurements were performed at a nominal power of 800 W and a wavelength of 10.6 μm using CO_2_ lasers. Excellent measurement agreement (1.02 %) was demonstrated, which was well within each of the expanded uncertainties from the two laboratories involved in this comparison.

## 1. Introduction

In the 1970s, the U.S. Air Force (AF) funded NIST (formerly NBS) to develop two primary reference standards for the measurement of high-power laser radiation emitted by the extremely large lasers being developed by the Department of Defense as potential weapons systems. These lasers were being designed to produce hundreds of kilowatts or more of CW (continuous wave) power and, at that time, no accurate measurement standards were available for measuring their power output. Consequently, the AF funded NIST to design and construct two large, electrically calibrated calorimeters to be used to measure the output power of the large lasers and to calibrate other detectors being used for this purpose.

In 1973, NIST scientists finished the design and construction of the BB1 [1] calorimeter for measuring the output of lasers having powers up to 100 kW. In 1978, NIST finished construction and testing of a second calorimeter, BB2 [2], which was similar to BB1 but had some additional improvements. After thorough testing and characterization, both calorimeters were delivered to the AFPSL, where they have been maintained and operated since that time. While both calorimeters are large (approximately 400 kg each), they are transportable and are carried in a customized, instrumented trailer when used for remote calibrations at high-energy laser sites around the country.

In 1999, the BB1 calorimeter was sent back to NIST for general refurbishment and electronic system upgrade. At the same time, engineers at NIST also made two major modifications to the system: (1) an external cooling water loop was added to allow the calorimeter to be cooled more quickly and easily, and (2) sensors from the backscatter and spillover monitors were modified to extend the wavelength capability of the calorimeter. A year later, the BB2 calorimeter was sent back to NIST for the same refurbishment, upgrade and modifications. All work on both calorimeters was completed in 2001. Since the modifications were quite extensive, a comparison between the NIST and the AF standards was deemed advisable to confirm that the AF standards (and the associated measurement system) were still in agreement with those of NIST.

## 2. Comparison Procedure

Due to the physical sizes of the BB1 and BB2 calorimeters compared to the space limitation of the NIST high-power laser laboratory, we could not directly compare them with the NIST high-energy standard calorimeter; consequently, we decided to conduct the comparison using a transfer standard. The transfer standard was a commercially available, water-cooled, absorbing-disc type power meter. The meter had been thoroughly studied at NIST for the past ten years and found to have a good repeatability and spatial uniformity. Additionally, the nonlinearity of the detector is small and has been well characterized. The comparison process consisted of three primary steps. First, the transfer standard responsivity (in units of mV/W) was calibrated at the NIST high-power standard Laboratory. Next, it was sent to AFPSL for calibration. Finally, the transfer standard was returned to NIST where it was then recalibrated and the final NIST calibration result was determined by averaging the two individual NIST calibration results.

## 3. NIST Measurement System

**Fig. 1:**
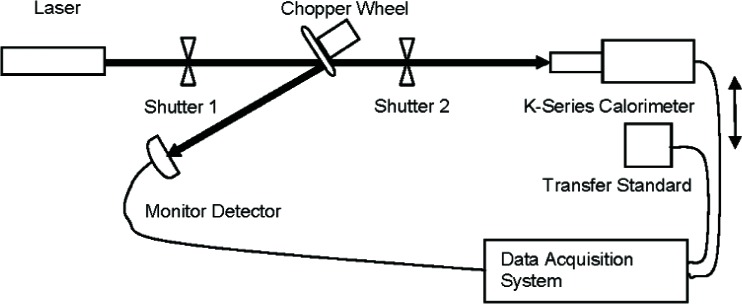
NIST Measurement System.

The NIST high-power CO_2_ laser measurement system (Fig. 1) consists of a high power standard calorimeter (designated as a K-series calorimeter [3]), a beamsplitter, a monitor detector, a CO_2_ laser, two optical shutters, and a data acquisition system.

The K-series calorimeter is an electrically calibrated calorimeter, which is capable of energy measurements in the range 300 J to 3000 J. It is typically used to measure CW lasers radiation at powers of 5 W to 1000 W for wavelengths of 1.06 μm and 10.6 μm. This calorimeter has been in constant use as a standard over the past forty years and has shown little or no change in response characteristics during this time period. An optical chopper wheel with reflecting blades was used as a beamsplitter, and a suitable power meter was used as a monitor detector to detect any variation in laser power during the measurements.

The NIST calibration procedure entailed two primary steps. First, with shutter 2 closed, shutter 1 was opened, exposing the monitor detector to the laser beam and allowing it to reach a steady-state condition (i.e., waiting for a period seven times the time constant of the detector). Then, shutter 2 was also opened, exposing the K-series calorimeter to the beam. After the desired time interval (chosen to keep the total absorbed energy within the operational limits of the K calorimeter), both shutters were closed simultaneously. Thus, for the time period that shutter 2 was open, the K-series calorimeter recorded the total energy in the transmitted laser beam and the monitor detector recorded average power in the reflected laser beam. Since this time interval was accurately measured, the average power in the transmitted beam could be calculated. In the second step, the K-series calorimeter was replaced by the transfer standard and the two shutters were opened simultaneously. After waiting for both detectors to reach a steady-state condition (i.e., waiting for a period seven times the time constant of the slowest detector), the average power of each detector output was recorded. By combining the two sets of measurement data (using the monitor detector output to correct for any power variations during the process), the responsivity of the transfer standard was calculated. This pair of steps was performed multiple times and an average responsivity was calculated.

## 4. AFPSL Measurement System

**Fig. 2:**
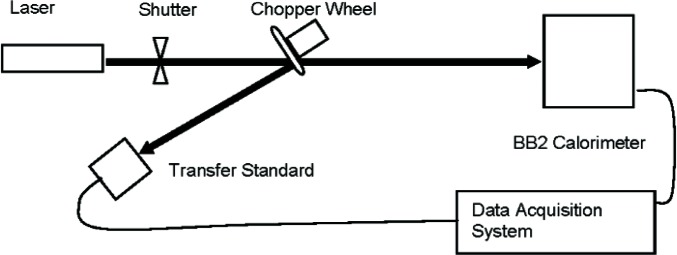
AFPSL Measurement System.

The AFPSL high-energy laser measurement system (Fig. 2) consists of a BB2 calorimeter, a CO_2_ laser, a beamsplitter, an optical shutter and a data acquisition system.

The BB1 and BB2 calorimeters are the electrically calibrated calorimeters with the enclosed cooling water circulation systems. The BB1 calorimeter measures energy in the range 30 kJ to 6 MJ using CW lasers having power outputs in the range 200 W to 100 kW, and the BB2 calorimeter measures energy in the range 30 kJ to 2.5 MJ using CW lasers having power outputs in the range 200 W to 100 kW.

A gold-plated chopper wheel with reflective blades is used as the beamsplitter. The beamsplitter ratio is periodically calibrated using a pair of commercial power meters. For this comparison, the transfer standard and BB2 were exposed simultaneously to the reflected and transmitted laser beams, respectively, from the beamsplitter. The exposure period, controlled by opening and closing the shutter, was chosen such that the energy incident onto BB2 was within its operational limits. The responsivity of the transfer standard was then calculated by dividing the integrated voltage recorded from the transfer standard by the incident energy measured by BB2. This process was repeated multiple times and an average responsivity was found for the transfer standard.

## 5. Results of Comparison

Table 1 and 2 list the source of uncertainties for the NIST [4] and AFPSL measurements, and Table 3 lists the relevant measurement conditions associated with the comparison and the calculated results for both laboratories. The uncertainty estimates for the NIST and AFPSL laser energy measurements were assessed by use of NIST recommended guidelines [5]. The relative expanded uncertainties (*k* = 2) of the transfer standard responsivities measured at NIST and AFPSL were respectively 1.2 % and 3.5 %. A small nonlinearity correction factor [6] was applied to the responsivity measured by the AFPSL to account for the slightly different power levels used.

## 6. Conclusion

The relative difference of 1.02 % between calibration responsivities found by the two laboratories (NIST and AFPSL) in this comparison was well within the associated combined measurement uncertainties. Consequently, this result confirms that the two high-energy laser primary calorimeters and their corresponding measurement systems are essentially in agreement within experimented uncertainty. Thus, the modifications performed by NIST on BB2 presumably had no detrimental effect on their accuracy.

## Figures and Tables

**Table 1:** Components of relative uncertainty for the NIST measurements, *k* = 1

	Type A %	Type B %
Test meter calibration (first time)	0.033	
Beamsplitter ratio (first time)	0.039	
Test meter calibration (second time)	0.032	
Beamsplitter ratio (second time)	0.082	
Optical shutter	0.001	
Linearity measurements	0.028	
Calorimeter inequivalence		0.144
Calorimeter absorptivity		0.341
Calorimeter heater leads		0.196
Calorimeter electronics		0.058
Calorimeter electrical calibration	0.010	
Laser/system instability (absolute calibration)		0.289
Laser/system instability (linearity measurement)	0.289	
Polynomial truncation		0.058
Attenuator ratio (absolute calibration)		0.090
Attenuator ratio (linearity measurement)		0.090

**Table 2:** Components of relative uncertainty for the AFPSL measurements, *k* = 1

	Type A %	Type B %
Digital voltmeter		0.058
Ref. Meter: BB2		1.732
Beamsplitter ratio		0.404
Counter/timer		0.058
Non-linearity		0.104
Test meter measurements	0.062	

**Table 3:** Comparison conditions and results

	NIST	AFPSL
Room temperature (ºC)	23	21
Transfer standard cooling water temperature (ºC)	24.3	23.4
Transfer standard cooling water flow rate (GPM)	1.0	1.2
Laser wavelength (μm)	10.6	10.6
Average laser power (W)	809	780
Laser beam size on the transfer standard (mm)	23	22.9
Average responsivity of the transfer standard (mV/W)	0.16035	0.16215
Nonlinearity corrected responsivity, referenced to 809 W (mV/W)	0.16035	0.16199
Relative expanded uncertainty (*k* = 2) (%)	1.2	3.5
Relative measurement difference [(AFPSL/NIST)–1] (%)	1.02	
